# Assessing the Predictive Validity of Risk Assessment Tools in Child Health and Well-Being: A Meta-Analysis

**DOI:** 10.3390/children12040478

**Published:** 2025-04-07

**Authors:** Ning Zhu, Xiaoqing Pan, Fang Zhao

**Affiliations:** 1 School of Social Development and Public Policy, Fudan University, Shanghai 200433, China; 2 Department of Social Sciences, University of Eastern Finland, 70211 Kuopio, Finland

**Keywords:** child health, child well-being, risk assessment tool, predictive validity, meta-analysis

## Abstract

Background/Objectives: Violence and harm to children’s health and well-being remain pressing global concerns, with over one billion children affected annually. Risk assessment tools are widely used to support early identification and intervention, yet their predictive accuracy remains contested. This study aims to systematically evaluate the predictive validity of internationally used child risk assessment tools and examine whether the tools’ characteristics influence their effectiveness. Methods: A comprehensive meta-analysis was conducted using 28 studies encompassing 27 tools and a total sample of 136,700 participants. A three-level meta-analytic model was employed to calculate pooled effect sizes (AUC), assess heterogeneity, and test moderation effects of tool type, length, publication year, assessor type, and target population. The publication bias was tested using Egger’s regression and funnel plots. Results: Overall, the tools demonstrated moderate predictive validity (AUC = 0.686). Among the tool types, the structured clinical judgment (SCJ) tools outperformed the actuarial (AUC = 0.662) and consensus-based tools (AUC = 0.580), suggesting greater accuracy in complex decision-making contexts. Other tool-related factors did not significantly moderate the predictive validity. Conclusions: SCJ tools offer a promising balance between structure and professional judgment. However, all tools have inherent limitations and require careful contextual application. The findings highlight the need for dynamic tools integrating risk and needs assessments and call for practitioner training to improve tool implementation. This study provides evidence-based guidance to inform the development, adaptation, and use of child risk assessment tools in global child protection systems.

## 1. Introduction

The risk of children suffering from various forms of harm to their health and well-being poses a persistent challenge to societal development and remains a major global concern. It is estimated that at least 1 billion children worldwide are exposed annually to one or more forms of violence, including corporal punishment, bullying, physical or emotional abuse, and sexual violence [1,2]. These risks—whether experienced in isolation or cumulatively—have profound and lasting impacts not only on children’s physical and mental health but also on their developmental trajectories. The consequences often extend into adulthood, potentially leading to cycles of harm across generations. Moreover, such forms of harm and abuse result in substantial global expenditures on judicial systems, healthcare, and social services, thereby negatively impacting socio-economic outcomes [3].

Violence against children is often hidden, typically occurring behind closed doors and rarely reported [1]. To identify and predict the likelihood of children experiencing harm—either currently or in the future—and to support child welfare professionals in making informed decisions that facilitate appropriate prevention or intervention efforts [4], risk assessment tools have become integral to child health and well-being practices [5]. These tools are designed to identify family risk factors, strengths, and available resources, thereby enabling investigations and referrals to be categorized according to varying levels of risk [6]. Notably, most tools fail to make a clear distinction between risk assessment and needs assessment [7]. At present, three primary categories of risk assessment tools are used in the field of international child health and well-being:

(a) Consensus instruments are developed by compiling and refining risk factors through expert analyses of various case types, resulting in a consensus-based checklist. These tools support child healthcare and well-being professionals in identifying both the conditions that contribute to harmful behaviors and the family strengths that enhance caregivers’ protective capacities [8,9,10]. Such assessments rely on the evaluator’s values, professional expertise, and capacity to integrate and apply knowledge to form subjective, descriptive judgments [11]. These tools are particularly valuable in addressing complex cases [12,13].
(b) Actuarial instruments are grounded in utility theory and rely on equations, formulas, charts, algorithms, or actuarial tables to generate graded estimates of the likelihood of harm to a child, typically expressed through standardized scoring systems [14,15,16]. These tools are valued for their predictive validity and their capacity to enhance consistency in decisions related to family intervention and service provision [17]. However, the predictive variables used in these instruments are derived from large-scale studies or meta-analyses, and their inclusion is contingent upon the quality and robustness of existing research [18].
(c) Structured clinical judgment (SCJ) instruments, a more recent development in the field, combine professional judgment methodologies to bridge the gap between clinical practice and scientifically grounded (actuarial) approaches to risk assessment [16,19]. These instruments are not direct hybrids of consensus- and actuarial-based tools; rather, they are purposefully designed to mitigate the limitations of both while preserving their respective strengths [20]. SCJ tools provide structured guidelines informed by the operationalization of variables across multiple dimensions. While some of these guidelines are empirically supported, the final decision-making authority remains with the practitioner [15,18]. Unlike actuarial instruments, the specific items included in SCJ tools are derived from comprehensive literature reviews rather than specific datasets [20].

Predictive validity is among the most critical criteria for evaluating the effectiveness of child risk assessment tools. Although international studies have empirically examined the predictive validity of individual instruments, systematic reviews and meta-analytic evaluations remain limited, and the existing findings are often inconclusive or contested. Compared to international practices, in many developing countries, risk assessment practices frequently rely on clinical experience, interview techniques, and practitioner intuition, highlighting a substantial gap in the adoption and validation of structured assessment tools. This study employs meta-analytic methods to evaluate the predictive validity of child risk assessment tools through a comprehensive review of relevant international research. It further examines whether specific tool characteristics—such as type, length, year of publication, assessor type, and target population—affect predictive accuracy. The findings aim to offer evidence-based insights that can support the development and refinement of risk assessment tools and practices within the field of child health and well-being.

## 2. Literature Review and Framework

Predictive validity, also referred to as predictive effectiveness, is the capacity to estimate the likelihood of future events or behaviors by identifying data trends, relationships, or patterns through statistical analysis [21]. In the context of child health and well-being, research focuses on whether assessment tools can accurately forecast the emergence of subsequent risks [22]. Over the past 70 years, the debate over the relative merits of actuarial methods versus clinical judgment has been a central theme in international research on risk assessment of child health and well-being. This ongoing discourse, often described as the “risk assessment wars” [23,24], has focused on which type of tool offers greater effectiveness in evaluating child risk.

One prevailing perspective holds that actuarial tools are superior to clinical judgment in risk assessment. Empirical research suggests that actuarial statistical predictions, even relatively simple statistical models, consistently outperform clinical judgments in terms of accuracy [8]. In a comprehensive review of 136 studies conducted between 1928 and approximately 2000, Grove and Meehl (1996) concluded that “the vast majority of studies favor actuarial methods [14]”. Similarly, D’Andrade et al. (2008), in their review of seven risk assessment tools, found that modern actuarial instruments demonstrate greater predictive validity than consensus-based tools [22]. Hanson and Morton-Bourgon (2009) also observed that empirically derived actuarial indicators are more accurate than unstructured professional judgments, while the accuracy of structured clinical judgment falls between that of actuarial indicators and unstructured clinical approaches [25]. However, they noted that the predictive validity of actuarial tools can vary depending on the specific issue being assessed and the characteristics of the sample. Van der Put et al. (2017) further affirmed that actuarial tools exhibit higher predictive validity compared to both consensus-based tools and structured clinical judgment tools [7].

An alternative perspective challenges the assumption that actuarial tools consistently outperform clinical judgment, suggesting that determining superiority may be inherently complex. For instance, Grove and Meehl (1996) identified studies in which clinical judgment outperformed actuarial models [14]. Similarly, Baumann et al. (2005) concluded that actuarial tools are not inherently superior to clinical approaches [26]. In a systematic review assessing the accuracy of 13 child risk assessment tools, Barlow et al. (2012) found that—apart from some supporting evidence for the California Family Risk Assessment (CFRA)—there is limited empirical support for the effectiveness of other tools in the field of child healthcare and well-being [15]. They further cautioned that the use of structured clinical judgment tools may have potential adverse consequences if implemented without adequate training, supervision, and professional support [15].

In a systematic review, Bartelink et al. (2015) [27] reported mixed findings regarding the effectiveness of risk assessment tools. While some studies suggested that actuarial tools outperformed clinical judgment, others found clinical judgment to be equally effective. However, this review was later critiqued by Van der Put et al. (2016) [28], who argued that its inclusion and exclusion criteria limited the scope of the literature under analysis. They contended that concluding that clinical judgment may equal or surpass actuarial methods is premature—if not erroneous—without a broader and more inclusive evidence base. In response, Bartelink et al. (2016) [29] clarified that they do not claim clinical judgment to be superior to actuarial methods. Instead, they emphasized that professionals must recognize the limitations of both their own clinical judgments and the tools they use [29]. Both actuarial and consensus-based tools have notable deficiencies in predicting child risk, necessitating caution in their application due to the current lack of robust empirical evidence. Supporting this perspective, Saini et al. (2019) [30] concluded that no single tool consistently outperforms others across different contexts and populations.

As McNellan et al. (2022) [31] noted, existing research on risk assessment tools exhibits demonstrates considerable heterogeneity, with variations in study design and methodological quality complicating the interpretation of findings. The current literature primarily comprises comparative studies that highlight the strengths of consensus-based and actuarial tools. Moreover, many studies rely on qualitative approaches to evaluate the predictive accuracy, underscoring the need for caution when adopting actuarial models in risk assessment, as these models do not always outperform clinical judgment [26]. Given these limitations, it is essential to undertake more extensive and in-depth quantitative research to determine which type of assessment tool offers a superior overall predictive validity. In addition, identifying the specific characteristics of these tools that enhance or hinder predictive performance will be critical to guiding the development and refinement of risk assessment tools, ultimately enhancing their practical effectiveness.

The inherent characteristics of risk assessment tools may significantly influence their predictive validity. First, the publication year of a tool may serve as a moderating role. Research indicates that the average publication year of studies assessing the effectiveness and reliability of such tools is 2006, rendering many potentially outdated and methodologically inconsistent [31]. Consequently, it is important to investigate whether a tool’s publication year impacts its predictive validity. Second, the type of tool—consensus-based, actuarial, or structured clinical judgment—may also affect the predictive validity. As previously discussed, ongoing debates regarding the superiority of different tool types remain unresolved, underscoring the need for further investigation into how the tool type influences risk prediction. Third, the number of items included in a tool may be another influential factor. Schwalbe’s (2007) analysis of juvenile justice risk assessment tools found that shorter tools are generally less effective at capturing relevant risk factors than longer ones [32], suggesting that the number of items may impact the predictive validity.

**Figure 1:**
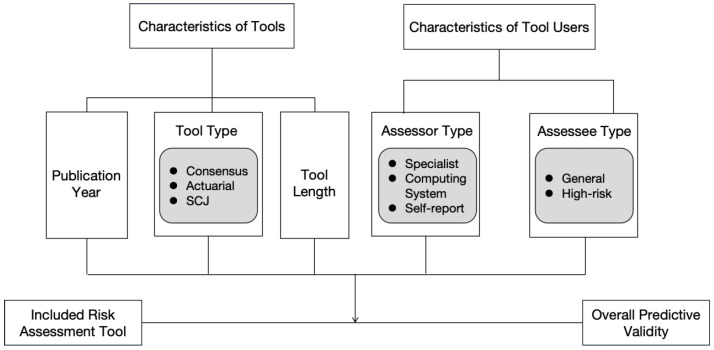
Analytical framework.

Secondly, the characteristics of tool users may also influence the predictive validity. This study primarily investigates the impact of user type and the subjects of assessment tools on the predictive validity. The user type, referring to the assessors, may include professionals, computer systems, or individuals completing self-reports. While different types of assessors could potentially affect the predictive validity, empirical evidence remains limited. For example, van der Put et al. (2017) found that the predictive validity does not depend on the assessor type, but no other studies have produced conclusive findings on this issue [7]. The subject type, which refers to whether the tool is used for screening in the general population or assessing the recidivism risk in high-risk populations, may also shape the predictive validity. As Cash (2001) suggested, differences in the focus of assessment tools, such as the type of subjects being evaluated, can result in variations in the predictive effectiveness [9]. The analytical framework of this study is presented in Figure 1.

## 3. Methods and Materials

This study employs meta-analysis, a quantitative review method grounded in large sample data. Meta-analysis embodies the principles of evidence-based research, aiming to extract, calculate, and synthesize numerical relationships among variables reported in prior studies in order to correct for biases present in the existing research [33]. Compared to the inherent subjectivity of qualitative literature reviews, meta-analysis offers a more objective, accurate, and replicable approach, making a significant advancement in the pursuit of empirical rigor within the social sciences. Globally, a wide range of child risk assessment tools exists; however, substantial disagreement persists among researchers regarding their predictive validity in assessing children’s potential risks. As a highly flexible methodology, meta-analysis provides new quantitative evidence on the predictive validity of these tools. Moreover, it offers critical insights and practical guidance for the theoretical development and refinement of child risk assessment instruments. Therefore, we followed the meta-analytic procedures outlined by van der Put et al. (2017) [7], whose work served as a key methodological reference for effect size calculation, conversion methods, and multilevel modeling.

### 3.1. Data Collection

The quality and quantity of literature included in a meta-analysis directly influence the reliability and validity of its results. Therefore, conducting a rigorous, standardized, comprehensive, and systematic literature search is essential to ensure analytical accuracy. This study conducted a systematic search across widely used international electronic databases, including the Web of Science Core Collection, BIOSIS Previews, Chinese Science Citation Database, Derwent Innovations Index, KCI-Korean Journal Database, MEDLINE, and SciELO Citation Index.

**Figure 2:**
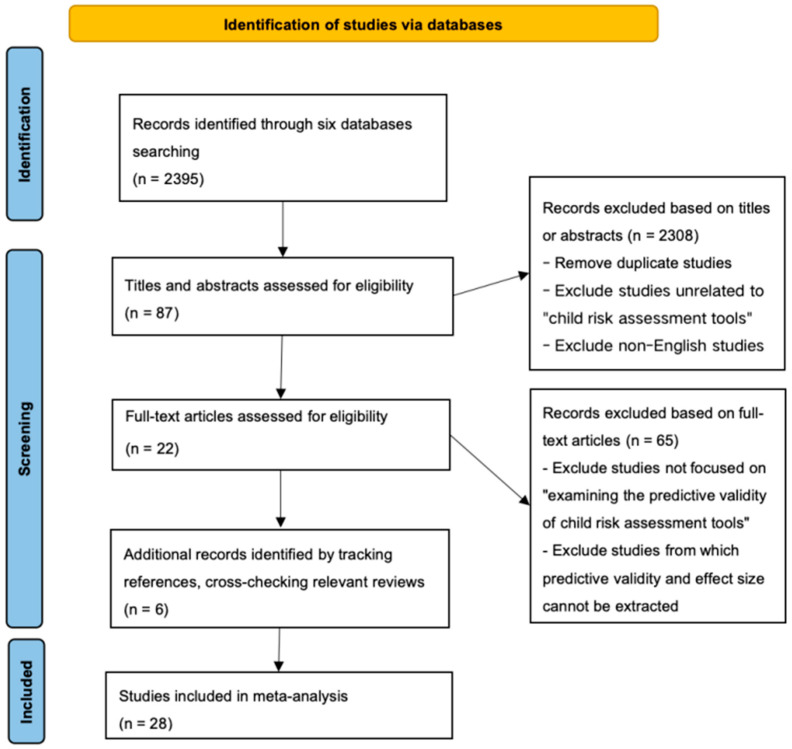
Illustration of the search results and the procedures for article selection.

Keywords were grouped into the following categories: risk assessment-related terms (e.g., risk assessment, risk tool, risk measure, risk evaluate, risk analysis, risk management, risk model, screening, and risk examination); abuse- and neglect-related terms (e.g., abuse, maltreatment, neglect, harm, and abandon); child protection-related terms (e.g., child protect and safeguard); child-related terms (e.g., child, infant, baby, toddler, teenager, adolescent, minor, and newborn); and predictive validity-related terms (e.g., AUC, ROC, sensitivity, specificity, predictive validity, and predictive accuracy). The logical relationship between abuse- and neglect-related terms and child protection-related terms was set as “OR”, while the relationships between the remaining keyword groups were set as “AND”. All keywords were searched within the “topic” field. The specific search strategy is illustrated in Figure 2. As of 31 December 2024, a total of 2395 articles were retrieved.

All retrieved literature was imported into Endnote for screening. Two researchers independently reviewed titles, abstracts, and keywords during the initial screening phase. Exclusion criteria at this stage included the following: removal of duplicates, exclusion of studies unrelated to “child risk assessment tools”, and removal of non-English publications. This process resulted in 87 potentially relevant studies. The researchers then independently read the full texts, excluding studies that did not evaluate the predictive validity of child risk assessment tools or lacked extractable data on predictive validity or actual effect sizes. This process yielded 22 studies.

An additional six English-language studies were identified through reference tracking, cross-checking relevant systematic reviews, and verifying data from existing meta-analyses. In total, 28 studies were included in the final analysis, with a combined sample size of N = 136,700. A detailed flow of the screening process is illustrated in Figure 2.

### 3.2. Coding and Quality Assessment

In accordance with standard meta-analytic procedures, a coding sheet was developed, and data from the final set of included studies were systematically recorded. The coding sheet captured variables such as author information, year of publication, tool name, tool type, tool length, assessor type, assessment target, sample size, correlation coefficient, standard error, mean, and standard deviation. To minimize the risk of coding errors, a double-coding approach was adopted: two researchers independently coded the data, and a professor—serving as a senior academic advisor with expertise in child welfare, child risk assessment, literature reviews, and evidence synthesis—helped mediate discrepancies during the literature screening and coding processes until full consensus was reached.

The methodological quality of the included studies was assessed using the CASP (Critical Appraisal Skills Programme) checklist, developed by the Centre for Evidence-Based Medicine at the University of Oxford. This tool for appraising the quality of diagnostic test studies comprises 12 items divided into three sections. The first section evaluates the reliability of study findings through six questions, including “Did the study define a clear research question?”, “Was the test being evaluated compared with an appropriate reference standard?”, and “Were all participants subjected to both the index test and the reference standard test?” The second section assesses the results and the confidence in those results through two questions. The third section evaluates the applicability of the findings using four questions, including “Can the results be applied to the target population?”, “Can the test be used for the target population?”, and “Are all outcomes important to the target population considered?” [34]. Each item was rated as “yes” or “no”, with a maximum possible score of 12. Studies scoring 10 or above were classified as “good”, those scoring between 7 and 9 as “moderate”, and those scoring below 7 as “poor”. Following evaluation, 22 of the 28 included studies were rated as “good”, while the remaining 6 were rated as “moderate”.

### 3.3. Statistical Analysis Process

The statistical analysis process began with a descriptive analysis of the included studies, followed by an estimation of bias. The Area Under the Curve (AUC) was used as the effect size, as it is the most reported metric for evaluating the predictive validity of risk assessment tools and is considered the preferred measure for this purpose [35]. In the context of child risk assessment, the AUC represents the probability that a randomly selected child who has experienced risk will be rated in a higher-risk category than a randomly selected child who has not experienced risk. The AUC ranges from 0.500 (accuracy no better than random chance) to 1.000 (perfect discrimination). An AUC between 0.556 and 0.639 is considered a small effect size, between 0.639 and 0.714 is a medium effect size, and 0.714 or above is a large effect size [36]. For studies that did not report AUC values, conversion methods from van der Put et al. (2017) were applied to calculate the AUC values [7].

After calculating the AUC values, they were first converted into Pearson’s correlation coefficients using Ruscio’s (2008) formula [37]. Since Pearson’s correlations do not follow a normal distribution, they were subsequently transformed into Fisher’s z values for statistical analysis. After analysis, Fisher’s z values were converted back into Pearson’s correlations to facilitate interpretation of the results. A three-level meta-analysis model was then employed to analyze all effect sizes, modeling three sources of variance: Level 1 variance represented the sampling variance of the effect sizes, Level 2 variance captured the within-study variance of effect sizes, and Level 3 variance accounted for between-study variance.

An intercept-only model (i.e., without covariates) was initially constructed to estimate the overall effect size represented by the intercept. The heterogeneity of effect sizes was then tested to determine whether the model could be extended to include potential moderator variables, such as the influence of tool characteristics. The analysis was conducted using the “rma.mv” function in the metafor package within the R programming environment [7]. To estimate coefficients in the multilevel meta-analysis model, an optimization adjustment strategy was applied, using a t distribution to compute individual regression coefficients and confidence intervals. For models expanded to include categorical moderator variables with three or more levels, hypothesis testing followed an F distribution. Statistical significance was set at *p* < 0.05.

## 4. Results

### 4.1. Descriptive Analysis

This study included 28 publications (n = 28), most of which were published between 2000 and 2023. The studies were conducted in the United States (n = 13), the Netherlands (n = 9), Hong Kong (n = 2), the United Kingdom (n = 1), Canada (n = 1), New Zealand (n = 1), and Japan (n = 1). Collectively, they examined the predictive validity of 27 distinct child risk assessment tools. In terms of the tool type, 4 were consensus-based, 16 were actuarial, and 7 employed structured clinical or professional judgment. Regarding the assessor type, 19 tools were administered by professionals, 1 by a computer system, and 7 through self-report. As for the target population, nine tools were designed for general population screening. Across the 28 studies, a total of 64 effect sizes were extracted, each representing the discriminative accuracy of a specific risk assessment tool or statistical prediction model. These tools, in aggregate, were used to assess 136,700 children and their families (total sample size N = 136,700), with individual study samples ranging from 118 to 50,671 participants. Detailed results are presented in Table 1.

### 4.2. Bias Testing

**Figure 3:**
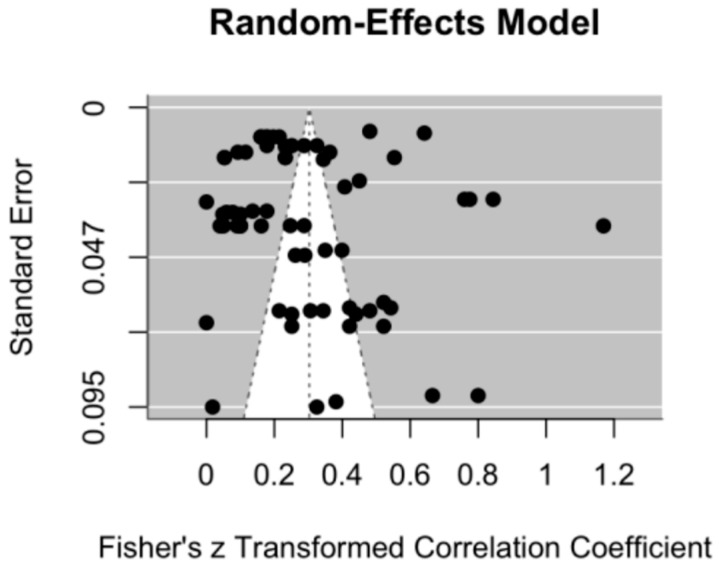
Funnel plot for publication bias testing.

The purpose of testing for publication bias in meta-analyses is to ensure that the included studies accurately reflect the broader research landscape, thereby mitigating biases that may arise from unpublished studies, small sample sizes, or the omission of studies with null findings. Such biases can distort the accuracy of meta-analytic conclusions [65]. This study employed Egger’s linear regression test and a funnel plot—two widely used methods—to assess potential publication bias. Egger’s test, originally introduced by Egger et al. (1997) [66], detects bias in meta-analyses through a linear regression approach. Specifically, the test regresses the standardized effect size (i.e., the effect size divided by its standard error) on the measure of precision (the inverse of the standard error). A statistically significant deviation of the intercept from zero (e.g., *p* < 0.05) indicates the presence of publication bias, whereas a non-significant *p*-value suggests its absence. In this study, Egger’s test yielded a *p*-value of 0.1202 for the main effect, indicating no statistically significant evidence of publication bias. Similarly, all the moderation effects also produced *p*-values greater than 0.05, including the tool type (*p* = 0.1528), tool length (*p* = 0.1204), publication year (*p* = 0.1545), assessor type (*p* = 0.0913), and target population type (*p* = 0.1341). These results suggest that publication bias was not a significant concern. The funnel plot further supported this conclusion, revealing no evidence of asymmetry or systematic bias (see Figure 3).

### 4.3. Main Effect Analysis

The statistical analysis revealed that the overall effect size for the predictive validity of the child risk assessment tools was z = 0.336 (SE = 0.038), t (64) = 8.747, *p* < 0.001, corresponding to an AUC of 0.686. These results indicate that the child risk assessment tools demonstrate statistically significant and moderately strong predictive validity, as presented in Table 2.

Regarding the heterogeneity of effect sizes, the one-sided likelihood ratio test for the second level of the three-level meta-analysis yielded a likelihood ratio value of 1265.0178 (*p* < 0.0001), while the third-level test produced a likelihood ratio value of 4.9635 (*p* = 0.0259). Both results were statistically significant, indicating a meaningful degree of heterogeneity in the effect sizes across different analytical levels.

These findings suggest the appropriateness of conducting a moderation analysis to examine whether specific characteristics of child risk assessment tools account for variance at the second and third levels. The aim of this analysis is to enhance the understanding of the predictive validity of these tools and to offer evidence-based insights for their further development. By investigating moderation effects, researchers can obtain a more nuanced understanding of how these tools perform across varying contexts, ultimately contributing to improved research design and more effective practical applications.

### 4.4. Moderation Effect Analysis

A series of bivariate models were conducted to evaluate the influence of each potential moderator on effect sizes. The results are summarized in Table 2. The moderators examined the following factors: (1) the tool type, categorized as consensus-based, structured clinical judgment, or actuarial; (2) the tool length, defined by the number of items included in the tool; (3) the year of publication; (4) the assessor type, classified as professional assessors, self-report, or computer-based; and (5) the target population type, categorized as either general or high-risk populations.

The moderation analysis revealed that the tool type had a statistically significant effect on the effect sizes. Specifically, the average effect size for the structured clinical judgment tools (AUC = 0.751) was significantly higher than that of the actuarial tools (AUC = 0.662) and consensus-based tools (AUC = 0.580). This finding suggests that structured clinical judgment tools may offer a superior predictive accuracy in assessing child risk compared to other tool types. Although the difference between the actuarial and consensus-based tools was not statistically significant, a trend toward significance was observed.

Further analyses examining the tool length, publication year, assessor type, and target population type as potential moderators did not yield statistically significant effects. These results suggest that, while such factors may influence the choice and implementation of assessment tools in practice, they do not appear to directly impact the predictive validity of these tools in child risk assessment.

## 5. Discussion

This study aimed to examine the overall predictive validity of child risk assessment tools and to assess whether the tool type, tool length, publication year, assessor type, or target population type influence the predictive accuracy. The results indicate that the child risk assessment tools included in this meta-analysis demonstrate moderate predictive validity (AUC = 0.686), suggesting that internationally utilized tools currently offer moderate accuracy in predicting child risk within health and well-being. This finding is consistent with the results of van der Put et al. (2017), who reported a comparable AUC value of 0.681 [7].

The moderation analysis identified the tool type as a significant moderating variable. The structured clinical judgment (SCJ) tools demonstrated a superior predictive validity (AUC = 0.751) compared to that of the actuarial tools (AUC = 0.662) and consensus-based tools (AUC = 0.580). This finding aligns with prior research in the field of violence risk assessment, which underscores the enhanced predictive validity of SCJ tools [20]. These tools incorporate empirically validated items, promoting standardization and improving inter-rater reliability [67]. Similarly, child protection instruments developed using highly structured methodologies share these benefits [15].

Although actuarial tools appeared to outperform consensus-based tools, this difference was not statistically significant, diverging from earlier studies that generally support actuarial tools for their higher predictive validity. This inconsistency warrants caution in adopting actuarial tools or models for risk assessment in child health and well-being contexts [26]. As previously discussed, ongoing debate persists within the academic community regarding whether actuarial tools genuinely provide a superior predictive accuracy [68].

Some scholars argue that actuarial tools often serve to replace, rather than support, the decision-making processes of practitioners and service users, potentially creating a sense of detachment from clinical judgment and frontline experience. Moreover, decisions derived from actuarial tools may be difficult to interpret, and the inherent vulnerabilities of big data—such as the susceptibility to errors, unreliability, and algorithmic biases—may result in flawed assessments or exacerbate existing biases, ultimately leading to unfair treatment for service recipients [68,69]. Research has also emphasized that actuarial tools typically fail to account for the contextual dimensions of risk and do not provide a framework for addressing client needs following assessment [70], emphasizing their limitations.

At the same time, although the SCJ tools demonstrated a better predictive validity than the actuarial and consensus-based tools, this does not imply they can be implemented in practice without careful consideration. It is essential to acknowledge the limitations inherent in all types of assessment tools. For instance, as previously discussed, actuarial tools have significant shortcomings. Consensus-based tools are similarly constrained by conceptual ambiguity, inconsistency in variable selection, reliance on a fixed set of predictors for a wide range of risk behaviors, limited focus on recurrence, and an overall modest predictive accuracy [71]. SCJ tools, while structured, may incorporate variables that are either unrelated or only weakly related to actual risk, thereby increasing the likelihood of inaccurate assessments [72].

### 5.1. Implications for Practice

This study offers several practical implications for the field of child protection. First, the structured clinical judgment (SCJ) tools demonstrated a superior predictive validity and should be prioritized in practice, particularly in complex cases that require professional discretion. While certain tool characteristics showed no significant effect on the predictive accuracy, practical considerations—such as assessor expertise, tool adaptability, and usability—remain important. Practitioners are encouraged to select and adapt tools based on specific service contexts and resource conditions.

Moreover, incorporating dynamic risk assessment and regular reassessment mechanisms can enhance ongoing risk monitoring. In developing regions where assessment systems are still emerging, high-performing tools identified in this study may serve as a foundation for localized adaptation and validation. Strengthening practitioner training and promoting policy-level support for standardized procedures and inter-agency collaboration will further advance the effectiveness and sustainability of child protection systems.

### 5.2. Strengths and Limitations of This Study

This study makes a significant contribution to the literature as one of the few meta-analyses to systematically examine the predictive validity of child risk assessment tools across diverse international contexts. By employing a three-level meta-analytic model, the study enhances analytical precision, allowing for the partitioning of sampling variance, within-study variance, and between-study variance. The inclusion of moderator analyses provides additional insights into the contextual and methodological factors that may influence tool performance.

Several limitations, however, must be acknowledged. First, the meta-analysis relied exclusively on published studies, which increases the risk of publication bias, despite the use of statistical methods to assess and control for it. Second, substantial heterogeneity across studies—in terms of the tool type, population characteristics, settings, and measurement approaches—may affect the comparability and consistency of effect sizes. Although the multilevel model helps address this variation, it cannot fully eliminate underlying differences in study quality or context. Third, the use of the Area Under the Curve (AUC) as the sole effect size, while widely accepted in predictive validity research, may not fully capture important dimensions such as sensitivity, specificity, or practical utility in real-world settings. This reliance may constrain the interpretability of the findings, especially for practitioners seeking to make context-specific decisions. Moreover, the tools examined in the included studies predominantly focus on static risk factors, which limits their capacity to capture dynamic, time-sensitive aspects of child and family circumstances. The absence of longitudinal measures or dynamic adjustment mechanisms in most tools further reduces their usefulness in ongoing monitoring and intervention planning. Additionally, some tools may have been validated in narrowly defined populations or regions, raising concerns about their cross-cultural applicability and generalizability.

Taken together, these limitations suggest that while the findings offer valuable synthesized evidence, they should be interpreted with caution and adapted thoughtfully when applied in practice. Future research should aim to include unpublished or gray literature, explore dynamic and context-sensitive indicators of risk, and incorporate multiple dimensions of predictive performance. Such efforts will help advance both the scientific understanding and the practical implementation of risk assessment in child protection systems.

## 6. Conclusions

This meta-analysis examined the predictive validity of 27 child risk assessment tools across 28 studies, providing the comprehensive syntheses to date in the field of child risk assessment. Overall, the findings indicate that these tools demonstrate a moderate predictive validity, with structured clinical judgment (SCJ) tools showing a superior performance compared to that of actuarial and consensus-based tools. The results suggest that SCJ tools may offer a more balanced approach by combining empirical structure with professional discretion, making them particularly valuable in complex and dynamic child welfare contexts.

However, other tool-related factors—such as tool length, publication year, assessor type, and target population—did not significantly influence the predictive validity. Nonetheless, these characteristics may still affect implementation and effectiveness in real-world settings. Practitioners should therefore consider not only statistical performance, but also contextual usability, cultural relevance, and training demands when selecting or applying tools. Moreover, while the SCJ tools showed relatively strong predictive accuracy, caution is warranted in their application, as all models have inherent limitations. For example, actuarial tools may overlook contextual nuances, whereas SCJ tools may include variables only weakly linked to actual risk. Many tools also emphasize identifying risk severity and intervention urgency but fall short in providing actionable guidance for tailored practice.

Future research should prioritize the development of dynamic assessment tools that integrate both risk and needs dimensions, thereby improving their ability to guide targeted intervention strategies. For developing countries still in the early stages of child risk assessment research and implementation, the findings suggest that localized tools may be adapted from internationally validated SCJ models—provided they undergo rigorous, evidence-based validation and contextual refinement. Additionally, training professionals with expertise in risk assessment remains essential. Advancing both tool development and professional capacity will better equip policymakers and practitioners to identify high-risk children, assess the likelihood of harm, and implement timely, appropriate prevention or intervention measures, ultimately safeguarding children’s rights and strengthening integrated systems of child health and well-being.

## Figures and Tables

**Table 1:** Basic characteristics of included studies.

Study	Tool Abbreviation Name	Tool Type	Tool Length	Assessor Type	Assessee Type	Sample Size
Van der Put 2023 [38]	SPARK	SCJ	24	Specialists	General Groups	1582
Day 2023 [39]	TeenHITSS	Actuarial	5	Self-report	General Groups	251
Day 2023 [39]	CTSPC	Actuarial	22	Self-report	General Groups	251
Moon 2022 [40]	AAPI–2	Consensus	40	Self-report	High-risk Groups	218
Vial 2021 [41]	ARIJ	Actuarial	30	Specialists	High-risk Groups	3681
Ruiter 2020 [42]	CARE-NL	SCJ	18	Specialists	High-risk Groups	211
Schols 2019 [43]	ERPANS	Actuarial	31	Specialists	General Groups	1257
Evans 2019 [44]	FACE-CARAS	Actuarial	48	Specialists	High-risk Groups	123
Lo 2017 [45]	RAM	SCJ	15	Specialists	High-risk Groups	265
Van der Put 2017 [46]	IPARAN	Actuarial	16	Self-report	General Groups	4692
Schouten 2017 [47]	SPUTOVAMO-R2/R3	Actuarial	5	Self-report	General Groups	50,671
Van der Put 2016 [48]	CFRA	Actuarial	25	Specialists	High-risk Groups	491
Horikawa 2016 [49]	PMCTR-J	Actuarial	6	Specialists	High-risk Groups	716
Van der Put 2016 [50]	CLCS	SCJ	75	Specialists	High-risk Groups	3963
Johnson 2015 [51]	CFRA	Actuarial	25	Specialists	General Groups	236
Dankert 2014 [52]	CFRA	Actuarial	25	Specialists	High-risk Groups	11,444
Vaithianathan 2013 [53]	PRM	Actuarial	132	Computing System	High-risk Groups	17,396
Coohey 2013 [54]	CFRA	Actuarial	21	Specialists	High-risk Groups	6832
Staal 2013 [55]	SPARK	SCJ	16	Specialists	General Groups	1850
Chan 2012 [56]	CARAS	Actuarial	64	Self-report	General Groups	2363
Ezzo 2012 [57]	C-CAPS	Actuarial	40	Specialists	High-risk Groups	118
Baumann 2011 [58]	CGRA	SCJ	77	Specialists	High-risk Groups	1199
Johnson 2011 [59]	CFRA	Actuarial	20	Specialists	High-risk Groups	6543
Barber 2008 [60]	ORA	Consensus	22	Specialists	High-risk Groups	1118
Sledjeski 2008 [61]	CT	Actuarial	24	Specialists	High-risk Groups	244
Ondersma 2005 [62]	CAP	Actuarial	160	Self-report	High-risk Groups	713
Loman 2004 [63]	M-SDM FRA	SCJ	25	Specialists	High-risk Groups	15,100
Chaffin 2003 [64]	CAP	Actuarial	160	Self-report	High-risk Groups	459
Baird 2000 [8]	SDM	Actuarial	NR	Specialists	High-risk Groups	929
Baird 2000 [8]	WRAM	Consensus	NR	Specialists	High-risk Groups	908
Baird 2000 [8]	CFAFA	Consensus	NR	Specialists	High-risk Groups	876

Note: SCJ = structured clinical judgment; NR = not reported. For tool full name, see Appendix A.

**Table 2:** Meta-analysis and overall effect size of predictive validity.

Moderating Variables	N	Effect Size (N)	Mean Fisher’s z (95% CI)	SE	Mean AUC	F (df1, df2)	*p*	Level 2	Level 3
Overall effect	28	65	0.336 (0.259, 0.412)	0.038	0.686		<0.001		
Tool type						5.499	0.006 **	0.043	0.002
Actuarial	19	44	0.291 (0.223, 0.359)	0.034	0.662				
SCJ	10	13	0.463 (0.323, 0.603)	0.070	0.751				
Consensus	4	8	0.142 (−0.029, 0.313)	0.086	0.580				
Tool length	28	65	0.341 (0.339, 0.343)	0.001	0.689	1.927	0.170	0.031	0.028
Publication year	28	65	0.399 (0.387, 0.411)	0.006	0.719	0.943	0.335	0.036	0.019
Assessor type						0.215	0.807	0.035	0.023
Specialists	24	48	0.339 (0.155, 0.523)	0.092	0.687				
Self-report	7	16	0.316 (0.156, 0.475)	0.080	0.675				
Computing system	1	1	0.481 (−0.026, 0.989)	0.254	0.760				
Assessee type						0.684	0.411	0.035	0.021
General	8	11	0.392 (0.235, 0.549)	0.078	0.715				
High-risk	21	54	0.318 (0.138, 0.497)	0.090	0.676				

Notes: ** = *p* < 0.01.

## Appendix A

The appendix shows the full names of all instruments included in this study.

**Table A1:** Full name of all instruments included in this study.

Tool Abbreviation Name	Tool Full Name
AAPI–2	The Adult-Adolescent Parenting Inventory–2
ARIJ	Actuarial Risk Assessment Instrument for Child Protection
CAP	Child Abuse Potential Inventory
CARE-NL	Child Abuse Risk Evaluation—Dutch version
CARAS	Child Abuse Risk Assessment Scale
C-CAPS	Cleveland Child Abuse Potential Scale
CFAFA	California Family Assessment Factor Analysis
CFRA	California Family Risk Assessment
CGRA	Concept-guided risk assessment
CLCS	Check List of Child Safety
CT	Connecticut risk assessment
CTSPC	Parent-Child Conflict Tactics Scales
ERPANS	Early Risks of Physical Abuse and Neglect Scale
FACE-CARAS	Functional Analysis in Care Environments-Child and Adolescent Risk-Assessment Suite
IPARAN	Identification of Parents at Risk for child Abuse and Neglect
M- SDM FRA	Minnesota SDM Family Risk Assessment
ORA	Ontario’s risk assessment tool
PMCTR-J	Prediction model for child maltreatment recurrence in Japan
PRM	Predictive risk model
RAM	Risk Assessment Matrix
SDM	Michigan Structured Decision-Making System’s Family Risk Assessment of Abuse and Neglect
SPARK	Structured Problem Analysis of Raising Kids
SPUTOVAMO-R2/R3	SPUTOVAMO-R2/R3
TeenHITSS	Teen Hurt-Insult-Threaten-Scream-Sex
WRAM	Washington Risk Assessment Matrix
